# Expression of Mouse MGAT in *Arabidopsis* Results in Increased Lipid Accumulation in Seeds

**DOI:** 10.3389/fpls.2015.01180

**Published:** 2015-12-22

**Authors:** Anna El Tahchy, James R. Petrie, Pushkar Shrestha, Thomas Vanhercke, Surinder P. Singh

**Affiliations:** ^1^CSIRO Agriculture, Canberra, ACTAustralia; ^2^CSIRO Food and Nutrition, Canberra, ACTAustralia

**Keywords:** monoacylglycerol acyltransferase (MGAT), diacylglycerol, triacylglycerol biosynthesis, *Arabidopsis thaliana*, seed oil increase, acyltransferase, gene expression

## Abstract

Worldwide demand for vegetable oil is projected to double within the next 30 years due to increasing food, fuel, and industrial requirements. There is therefore great interest in metabolic engineering strategies that boost oil accumulation in plant tissues, however, efforts to date have only achieved levels of storage lipid accumulation in plant tissues far below the benchmark to meet demand. Monoacylglycerol acyltransferase (MGAT) is predominantly associated with lipid absorption and resynthesis in the animal intestine where it catalyzes monoacylglycerol (MAG) to form diacylglycerol (DAG), and then triacylglycerol (TAG). In contrast plant lipid biosynthesis routes do not include MGAT. Rather, DAG and TAG are either synthesized from glycerol-3-phosphate by a series of three subsequent acylation reactions, or originated from phospholipids via an acyl editing pathway. Mouse MGATs 1 and 2 have been shown to increase oil content transiently in *Nicotiana benthamiana* leaf tissue by 2.6 fold. Here we explore the feasibility of this approach to increase TAG in *Arabidopsis thaliana* seed. The stable MGAT2 expression resulted in a significant increase in seed oil content by 1.32 fold. We also report evidence of the MGAT2 activity based on *in vitro* assays. Up to 3.9 fold increase of radiolabeled DAG were produced in seed lysate which suggest that the transgenic MGAT activity can result in DAG re-synthesis by salvaging the MAG product of lipid breakdown. The expression of MGAT2 therefore creates an independent and complementary TAG biosynthesis route to the endogenous Kennedy pathway and other glycerolipid synthesis routes.

## Introduction

Demand for production and use of plant oils is rapidly increasing and current agricultural practices are unable to produce sufficient amounts cost-effectively. Multiple approaches are being employed to develop plants with altered oil content to meet this demand. One approach that biotechnologists are exploring is the potential of transgenic technologies to boost the metabolic flux of carbon into oil in the developing seed (Weselake et al., 2008; Bates et al., 2013). In eukaryotes, neutral lipid synthesis pathways play an important role in energy storage. Each step is catalyzed by multiple isoforms of enzymes that differ in tissue distribution and subcellular localization. In higher eukaryotes the most direct biochemical pathway leading to TAG biosynthesis is the acyl-CoA dependent Kennedy or glycerol phosphate pathway (Kennedy, 1961). Recent work, however, has demonstrated that TAG accumulation in plant cells is not necessarily as unidirectional as this traditional model indicates (Bates et al., 2007, 2009). Rather complex interchanges occur between different neutral lipid pools of the Kennedy pathway and polar membrane lipids. Weselake et al. (2008) reported that neutral lipid production is affected by the interaction of a growing suite of enzymes with intermediates of Kennedy pathway.

In plants DAG represents the immediate precursor of TAG and is synthesized mainly by: (1) Kennedy pathway for *de novo* DAG synthesis (Kennedy, 1961), and (2) transfer of phosphocholine head group from phosphatidylcholine (PC) to PC-derived DAG by phosphatidylcholine:diacylglycerol cholinephosphotransferase (PDCT; Lu et al., 2009). Most of the DAG produced by the Kennedy pathway is transiently converted to PC before it is used to make TAG (Bates et al., 2012). The conversion of MAG to DAG by MGAT has been well-studied in animals (Yen et al., 2002; Cao et al., 2003). The presence of MAG has been reported in plants (Perry and Harwood, 1993), and it is only known to act as a precursor for cuticular wax biosynthesis (Li-Beisson et al., 2010).

A number of studies have established that seed oil content can be increased by changing the expression levels of individual enzymes involved in oil metabolism (Roesler et al., 1997; Zou et al., 1997; Jako et al., 2001; Vigeolas et al., 2007; Kelly et al., 2013a; Kim et al., 2014). Recent studies have reported an enhancement in TAG accumulation when a bifunctional Oleosin enzyme (OLE3) that has both MGAT and Phospholipase activities was expressed in *Saccharomyces cerevisiae* (Parthibane et al., 2012). Also, the transient expression of the mouse MGATs (*MmMGAT1* and *2*) gene in *Nicotiana benthamiana* plant leaves was found to increase the leaf oil content to exceed those of leaves expressing *Arabidopsis thaliana* diacylglycerol acyltransferase (DGAT1) positive control (Petrie et al., 2012). MGAT1 and 2 transient expressions led to 9.2-fold and 7.3-fold increase in TAG respectively at 5 days post-infiltration (dpi) in the infiltrated leaves (Divi et al., 2014). Nevertheless, Divi et al. (2014) also reported senescence-like symptoms by 5 dpi that are specific to MGAT1 infiltration only.

Seed oil content is also controlled by the balance between synthesis and breakdown in many eukaryotes, and a deficiency in TAG hydrolysis has been shown to result in greater oil deposition (Zimmermann et al., 2004; Grönke et al., 2005; Kurat et al., 2006). Chia et al. (2005) have shown a loss of at least 10% of storage lipids in *Brassica napus* during the desiccation stage of seed development. Recently, Van Erp et al. (2014) have used *A. thaliana* as an experimental system, where they overexpressed WRINKLED1 (a transcriptional regulator of glycolysis and fatty acid synthesis) and DGAT1 combined with suppression of the TAG lipase SUGAR-DEPENDENT1 (SDP1). Higher percentage seed oil content was achieved by minimizing the lipid turnover and TAG degradation (Van Erp et al., 2014).

In seed tissue the contribution of MGAT2 toward lipid biosynthesis remains to be proven. Since evidence exists for *de novo* MAG production in plant tissue we explored the feasibility of salvaging this MAG pool as a substrate for TAG synthesis or resynthesis in seed (Reddy et al., 2010; Yang et al., 2010). *A. thaliana* has served as a good model to explore novel strategies for seed oil increase. This model system provides the ability to grow large number of plants under controlled light and temperature conditions generating better statistical data. Therefore, the stable expression of mammalian MGAT2 in *A. thaliana* was established in order to investigate its effect on seed oil accumulation. In this study we demonstrate a significant increase in the oil content in seeds expressing the MGAT2 gene. In addition, we provide biochemical evidence for a possible role of MGAT2 salvaging MAG that is generated during TAG degradation before seed maturity for DAG synthesis. This study explores an independent pathway to the endogenous Kennedy pathway in plant seed tissue that relies on MAG as an intermediate and offers potential applications for both food and fuel applications.

## Materials and Methods

### Plant Material and Growth Conditions

Hundred seeds of each wild type *Arabidopsis* (ecotype Columbia), vector-only control, and stably transformed MGAT2 expressing seeds were surface sterilized and plated on selective agar-solidified MS medium supplemented with 50 mg/L kanamycin. The plates were placed at constant temperature (24°C) and 16 h/8 h photoperiod (50 μmol m^−2^ s^−1^). After germination, seedlings were transplanted into soil (2 g mini osmocote/L Debco mix). Plants were grown at 16 h light (24°C)/8 h dark (18°C) either in glasshouse without artificial light (T_0_–T_2_) or in a growth cabinet (T_3_ and T_4_) with an average of 350 μmol m^−2^ s^−1^. Each plant was individually covered with sleeves to avoid cross contamination. Developing siliques where tagged after flowering and collected for *in vitro* assays. Mature seeds were collected for lipid analyses at senescence.

### Stable Transformation of *A. thaliana*

The stable transformation of *A. thaliana* seeds (ecotype Columbia) with *B. napus* napinFP1::Musmu-MGAT2-GFP was performed using the floral dip method (Clough and Bent, 1998). The coding region of the Geneart (Life Technologies, Regensburg, Germany) synthetic construct 0954364_Musmu-MGAT_pMA (NM_177448), contained within an *Eco*RI fragment, was inserted into a custom vector pJP3386 (Supplementary Figure S1) at the *Eco*RI site, creating pJP3390. The seeds from the transformed plant were viewed after maturation under a Leica MZFLIII dissection microscope coupled to 0.5x M-series objective, 1x C-mount adaptor, and 100-W mercury-vapor burner for fluorescence imaging (Leica Microsystems). Transgenic seeds (strongly GFP positive) and Non-transgenic (GFP negative) seeds were selected. Subsequent generations of transgenic *A. thaliana* plants expressing *FP1::MGAT2* were grown in a random design alongside vector-only pORE04 (AY562534-AY562548; *Arabidopsis* Biological Resource Centre) and Columbia (parental) controls as described above.

### Lipid Analysis

*Arabidopsis thaliana* seeds were dried in a dessicator for 24 h and approximately 4 mg of seed were transferred to 2 mL glass vials with Teflon-lined screw caps. Seventy (70) μg of triheptadecanoin (Nu-Chek PREP, Inc., USA) were added to the vial as internal standard. Seed fatty acid methyl esters (FAME) were prepared by adding 0.75 mL of 1 N methanolic HCl (Supelco, Bellefonte, PA, USA) to seed material. The mixture was vortexed briefly and incubated at 80°C for 2 h. After cooling to room temperature, 0.3 mL of 0.9% NaCl (w/v) and 0.3 mL hexane were added to the vial and mixed well for 10 min in a Heidolph Vibramax 110. The FAME was collected and analyzed by GC with a flame ionization detector GC-FID (7890A GC, Agilent Technologies, Palo Alto, CA, USA) equipped with a 30 m BPX70 column (0.25 mm inner diameter, 0.25 mm film thickness, SGE, Austin, TX, USA) as described previously (Zhou et al., 2011). Peaks were integrated with Agilent Technologies ChemStation software (Rev B.04.03). Total FAME (TFA) were quantified based on the internal standard addition and expressed as a percentage of the SW.

### *In vitro* MGAT Activity

Developing siliques were collected at late seed development stage. Siliques were ground at 4°C in a solution containing 0.1 M potassium phosphate buffer (pH 7.2) and 0.33 M sucrose using an Ultra-Turrax homogenizer (IKA T10 Basic S5, Staufen, Germany). The homogenate was centrifuged at 20,000 *g* for 45 min at 4°C after which the supernatant was collected. Protein contents of the lysates were measured using a Wallac1420 multilabel counter and a Bio-Rad Protein Assay dye reagent (Bio-Rad Laboratories, Hercules, CA, USA). MGAT assay was performed according to Cao et al. (2007) with some modifications previously reported in (Petrie et al., 2012). [^14^C]glycerol-labeled *sn*-2-monooleoylglycerol (55 mCi/mmol, American Radiochemicals, St. Louis, MO, USA) was purified on SIL G-25 TLC plate to remove any radiolabeled free fatty acid or DAG contamination. The assays were carried out for 5, 10, 15, and 30 min at room temperature with gentle mixing. The reaction was stopped by adding 300 μL of chloroform/methanol (2/1 v/v). Total lipids were extracted as described by Bligh and Dyer (1959). Reaction mixtures were centrifuged (2000 *g* for 5 min), the lower phase collected and the upper phase extracted a second time with 200 μL chloroform. Total lipid from the combined chloroform phases were separated on pre-coated SIL G-25 TLC plate (Macherey-Nagel, Germany) in hexane/diethyl ether/acetic acid (70/30/1, v/v/v). MAG and DAG fractions were identified by running appropriate standards alongside the assay extractions. The TLC plate was exposed to phosphor imaging screens overnight and analyzed by a Fujifilm FLA-5000 phosphorimager. The radioactivity of each MAG and DAG was measured using a Ready Safe liquid scintillation cocktail (Beckman-Coulter, Inc., 4300N, Fullerton, CA, USA) and Beckman-Coulter Tri-Carb 2810 TR scintillation analyzer (Perkin Elmer, Singapore).

## Results

### MGAT-Mediated Lipid Accumulation in *A. thaliana* Seed

**FIGURE 1:**
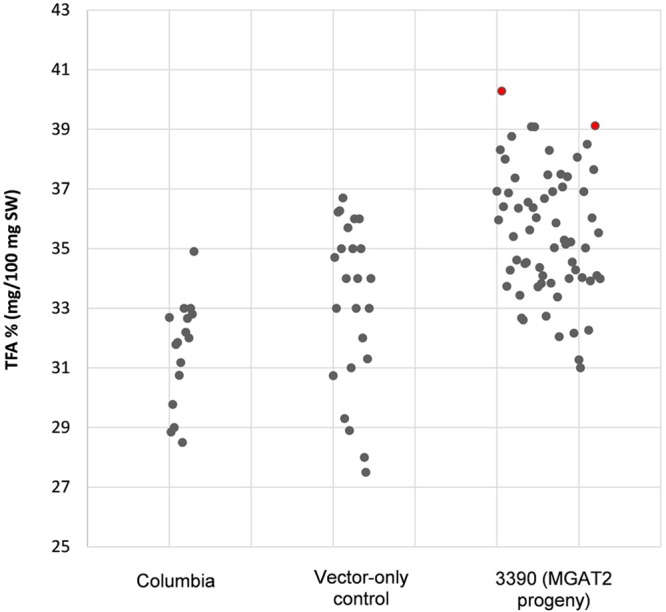
**Total fatty acid levels in stably transformed (3390) MGAT2 *Arabidopsis thaliana* T_2_ seeds relative to Columbia (parental) and vector-only control.** The average TFA level of 3390 (MGAT2 progeny) is significantly higher than Columbia and vector-only controls averages at *p* = 4.2E-06.

**FIGURE 2:**
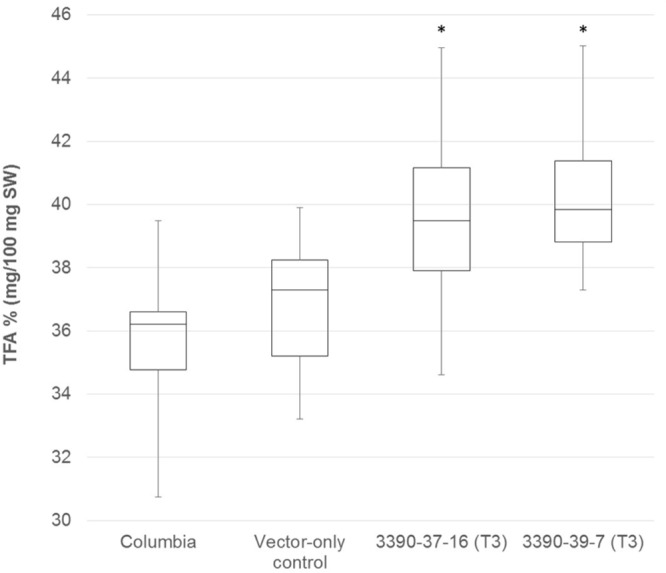
**Box-plot diagram showing the total fatty acid content in T_3_ MGAT2 lines 3390-37-16 (*n* = 25; median 39.5) and 3390-39-7 (*n* = 25; median 39.8) relative to Columbia (parental; *n* = 26; median 36.2) and vector-only control (*n* = 23; median 38.0).** ^∗^This is significantly different at *p* = 2.7E-07 (3390-37-16) and *p* = 9.4E-07 (3390-39-7) relative to Columbia and vector-only control.

The chimeric vector containing MGAT2 under the control of the seed specific truncated *B. napus* napin (FP1) promoter was used to transform *A. thaliana* by the floral dip method. T_1_ Seeds were plated onto medium containing kanamycin to select T_2_ transformed plants. Thirty plants were grown in a random design alongside vector-only and Columbia (parental) controls. T_2_ seeds were harvested and the total FAME (TFA) determined by direct methylation. We observed resulting TFA levels varying between 31 and 40.3% TFA SW. The average TFA level is significantly higher than Columbia and vector-only control averages (**Figure 1**). 3390-39 (40.3% TFA SW) and 3390-37 (39.1% TFA SW) lines were selected for further studies in a following generation. The TFA levels in the transgenic T_3_ seeds were found to be up to 1.45-fold increase over Columbia and vector-only control seeds. Kanamycin selection showed that T_3_ generation was segregating but the putative homozygotes 3390-39-7 and 3390-37-16 resulted in significantly elevated TFA content relative to Columbia and vector-only control (respectively, *F* = 32.4 and *F* = 36.7, *p* = 9.4E-07 and *p* = 2.79E-07, *F* crit = 4.06). TFA content in 3390-39-7 and 3390-37-16 seeds showed up to 10% relative increase (**Figure 2**). Vector-only control average showed slightly higher levels than Columbia (parental), nevertheless statistics showed no significant difference (*F* = 2.16, *p* = 0.14, *F* crit = 4.06). In addition there was no significant effect on the FA profile (**Table 1**). Oleic acid (18:1^Δ9^) and gondoic acid (20:1^Δ11^) levels were found to be slightly increased with a slight decrease in hexadecanoic (16:0) and linoleic acid (18:2^Δ9,12^) compared to control lines (**Table 1**).

**Table 1:** Major fatty acids of total lipids isolated from representative controls and transgenic MGAT2 *Arabidopsis thaliana* T_3_ seeds.

		14:0	16:0	16:1^Δ3t^	18:0	18:1^Δ9^	18:1^Δ11^	18:2^Δ9,12^	18:3^Δ9,12,15^	20:0	20:1	Other
Columbia	*n* = 25	0.1 ± 0.01	8.2 ± 0.03	0.4 ± 0.02	3.3 ± 0.16	12.8 ± 0.70	1.8 ± 0.09	28.4 ± 0.30	19.9 ± 0.60	2.2 ± 0.13	17.1 ± 0.50	5.9 ± 0.01
pORE04	*n* = 15	0.1 ± 0.02	8.1 ± 0.01	0.3 ± 0.01	3.3 ± 0.12	13.2 ± 0.70	1.8 ± 0.06	28.4 ± 0.30	20.0 ± 0.60	2.2 ± 0.07	17.2 ± 0.40	5.5 ± 0.01
MGAT2	*n* = 23	0.1 ± 0.01	7.4 ± 0.40	0.3 ± 0.03	3.0 ± 0.30	15.0 ± 0.10	1.5 ± 0.17	27.2 ± 0.19	19.3 ± 0.20	2.1 ± 0.80	19.0 ± 1.80	5.3 ± 0.01


### MAG Salvage by MGAT Acyltransferase in *A. thaliana* Developing Seed

**FIGURE 3:**
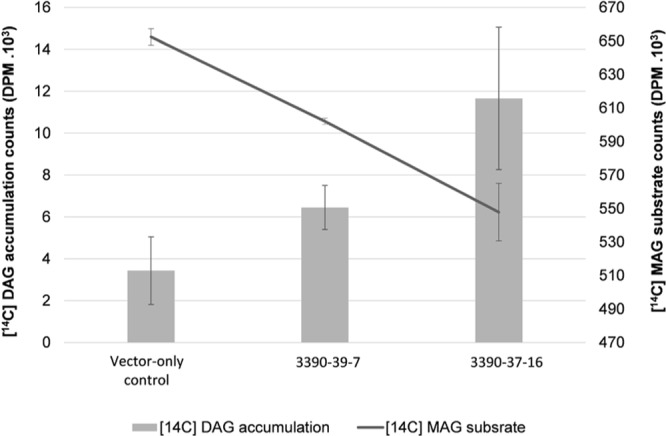
**Radioactivity [disintegrations per minute (DPM)] of MAG salvage and diacylglycerol (DAG) production in 3390-39-7 and 3390-37-16 developing silique lysates at 30 min of incubation with [^14^C] *sn*-2-MAG and unlabeled oleoyl-CoA**.

In order to further investigate the biochemical nature of the MGAT2 lipid accumulation response, we performed labeling studies on developing seeds. Lysate was prepared from a pool of developing siliques of 10 plants of the two 3390-39-7 and 3390-37-16 lines studied above that showed the highest lipid accumulation. [^14^C] *sn*-2-MAG and unlabeled oleoyl-CoA were added to each lysate sample followed by quantification of the labeled DAG reaction product at 5, 10, 15 (data not shown) and 30 min of incubation. Developing silique lysate of MGAT2 plants incorporated 17% of the labeled MAG substrate that was converted to DAG at 30 min of feeding (**Figure 3**). Labeled DAG accumulation exceeded that of the vector-only control by 1.9 and 3.4 fold in each line, respectively.

## Discussion

The simplest yet powerful method of economically feasible vegetable oil production is to increase its oil content by genetic manipulation of lipid biosynthetic pathways. Modifying oilseed crops to produce oils of uniform composition containing fatty acids varying in chain length or possessing reactive functional groups is a primary objective (Jaworski and Cahoon, 2003), as is that of increasing the yield of seed oil (Lardizabal et al., 2008; Zheng et al., 2008).

Oil increase in vegetative tissue via a MAG pathway was recently demonstrated by Petrie et al. (2012) and Divi et al. (2014). The authors showed that oil levels were increased 7.3-fold by transiently expressing MGAT2 in *N. benthamiana* leaves. Whilst the feasibility in a plant leaf system has been demonstrated, this concept can be applicable to any tissue that contains G-3-P and acyl-CoA substrates. DGAT catalyzes the acyl-CoA-dependent acylation of *sn-*1,2-DAG to produce TAG. DAG produced by MGAT2 may subsequently be converted to TAG by native mechanisms including DGAT activity, which may be upregulated in response to the DAG accumulation (Petrie et al., 2012), however, further investigation is required to confirm this. In this study DGAT activity may require longer time periods for the conversion of *de novo* DAG into TAG. On the other hand, a study has reported that the murine MGAT2 demonstrates a strong MGAT activity and a weak DGAT activity, with the latter being sensitive to inactivation with detergent (Cao et al., 2003). The DGAT activity is more pronounced with an extended incubation time. Furthermore, in their study, Cao et al. (2003) found that the DGAT activity is much weaker than the MGAT activity as assessed by the rate of formation of radioactive products catalyzed by mouse MGAT2 in the assay. The biosynthesis of DAG occurs mainly in the microsomal membranes through sequential acylation of G-3-P to TAG via DAG, unlike in the MAG pathway, where MAG is acylated to DAG by MGAT (Kennedy, 1961; Kayden et al., 1967).

Roesler et al. (1997) achieved 5% increase in seed oil content in the rapeseed by expressing a cytosolic version of the Acetyl-CoA carboxylase enzyme targeted to the rapeseed chloroplast. Cernac and Benning (2004) has reported that the expression of the WRINKLED1 transcription factor can have a “Push” effect on the glycolysis and fatty acid synthesis. Another study has shown that the expression of DGAT1 can also be used to “Pull” fatty acids into TAG (Jako et al., 2001). Finally, protecting TAG from lipolysis by expressing Oleosin genes has also been reported to minimize lipid turnover (Fan et al., 2013; Kelly et al., 2013b; Vanhercke et al., 2013; Winichayakul et al., 2013). Combining these gene modifications on seed oil content and yield in *A. thaliana* have been investigated. A combined WRINKLED1+DGAT1 stable expression in *A. thaliana* increased the percentage of seed oil content to approximately 44% SW, which is an approximately 1.2-fold increase over the wild type (Van Erp et al., 2014). These results are comparable to our findings. The highest lipid accumulating MGAT2 expressing line reported herein yielded 1.45 fold increase in the same plant model system. TFA percentage reached 45% TFA SW in 3390-37-16 and 3390-39-7.

On the other hand, the oil content is controlled by the balance between synthesis and breakdown in many eukaryotes, and a deficiency in TAG hydrolysis has been shown to result in greater oil deposition (Zimmermann et al., 2004; Grönke et al., 2005; Kurat et al., 2006). The breakdown of storage oil is initiated by lipases which catalyze the hydrolysis of TAG to release free fatty acids and glycerol (El-Kouhen et al., 2005; Quettier and Eastmond, 2009; Li-Beisson et al., 2010). Similarly, SDP1 and SDP1-LIKE encode a TAG lipase with a patatin-like acyl-hydrolase domain that can associate with the storage oil body surface and is capable of hydrolyzing TAG in preference to DAG or MAG (Eastmond, 2006; Kelly et al., 2011). Chia et al. (2005) have shown a loss of at least 10% of storage lipid in *B. napus* during desiccation stage of development. They have also shown that the activities of enzymes of the glyoxylate cycle, β-oxidation, and gluconeogenesis are detectable during the development of the *B. napus* embryo, including the period of oil accumulation, and that they increase as the embryo desiccates and matures. The 10–14% decrease in lipid content reported for glasshouse-grown *B. napus* is less than that for tobacco or *A. thaliana* grown in controlled environments, with respective decreases of 18 and 25% (Baud et al., 2002; Tomlinson et al., 2004). Other studies have also provided evidence that the turnover of fatty acids is a normal part of embryo metabolism in developing *B. napus* embryos where TAG is degraded during seed desiccation and maturation (Norton and Harris, 1975; Murphy and Cummins, 1989; Kang et al., 1994; Eastmond and Rawsthorne, 2000). In the present study, by expressing MGAT2 in seed we have explored the possibility of salvaging of *sn-2*-MAG before seed maturity. Further studies should be done to measure the breakdown of TAG into MAG. Due to *A. thaliana* seed size, this latter study will be done at different seed developmental stages in another oil crop like Canola where TAG breakdown has already been reported. We have introduced a new step in the acylglycerol pathway that acts on recycling the degraded TAG into DAG via MAG, hence the increase in oil yield in MGAT2 transgenic plants. This study is the first to report on the use of MAG as a substrate for *de novo* lipid biosynthesis in transgenic plant seed. No significant effect on the morphology and on seed germination was observed in the stably transformed lines generated in this study Supplementary Figure S2.

## Conclusion

We were able to demonstrate a novel lipid biosynthesis pathway in seed by expressing the mouse MGAT2 to salvage MAG and supply additional DAG for TAG production. By complementing the Kennedy pathway to increase the lipid yield we have demonstrated the feasibility of this system in oilseed. We were able to achieve up to 10% relative increase in the total oil content in MGAT2 transgenic seed. It will be interesting to determine whether a transgenic expression of MAG pathway in plants is subject to the same regulation as the endogenous Kennedy pathway and how it might interact with other transgenic strategies that can increase oil levels.

## Author Contributions

AElT conceived of the study, performed the experiments and drafted the manuscript. JP participated in plant transformation and in the drafting of the manuscript. PS performed the initial screening of T1 generation. TV participated in drafting and critical review of the manuscript. SS participated in conceptualization, and critical review of the manuscript.

## Conflict of Interest Statement

The authors declare that the research was conducted in the absence of any commercial or financial relationships that could be construed as a potential conflict of interest.

## Abbreviations

DAG: diacylglycerol
DGAT: diacylglycerol acyltransferase
G-3-P: glycerol-3-phosphate
MAG: monoacylglycerol
MGAT: monoacylglycerol acyltransferase
TAG: triacylglycerol
TFA: total fatty acid
SW: desiccated seed weight
